# Effects of human mobility and behavior on disease transmission in a COVID-19 mathematical model

**DOI:** 10.1038/s41598-022-14155-4

**Published:** 2022-06-27

**Authors:** Juan Pablo Gutiérrez-Jara, Katia Vogt-Geisse, Maritza Cabrera, Fernando Córdova-Lepe, María Teresa Muñoz-Quezada

**Affiliations:** 1 Centro de Investigación de Estudios Avanzados del Maule (CIEAM), 3480112 Talca, Chile; 2 Universidad Adolfo Ibáñez, Facultad de Ingeniería y Ciencias, 7941169 Santiago, Chile; 3 Universidad Católica del Maule, Vicerrectoria de Investigación y Postgrado, 3480112 Talca, Chile; 4 Universidad Católica del Maule, Facultad de Ciencias Básicas, 3480112 Talca, Chile; 5 Faculty of Health Sciences, Universidad Católica del Maule, 3480112 Talca, Chile

**Keywords:** Applied mathematics, Infectious diseases

## Abstract

Human interactions and perceptions about health risk are essential to understand the evolution over the course of a pandemic. We present a Susceptible-Exposed-Asymptomatic-Infectious-Recovered-Susceptible mathematical model with quarantine and social-distance-dependent transmission rates, to study COVID-19 dynamics. Human activities are split across different location settings: home, work, school, and elsewhere. Individuals move from home to the other locations at rates dependent on their epidemiological conditions and maintain a social distancing behavior, which varies with their location. We perform simulations and analyze how distinct social behaviors and restrictive measures affect the dynamic of the disease within a population. The model proposed in this study revealed that the main focus on the transmission of COVID-19 is attributed to the “home” location setting, which is understood as family gatherings including relatives and close friends. Limiting encounters at work, school and other locations will only be effective if COVID-19 restrictions occur simultaneously at all those locations and/or contact tracing or social distancing measures are effectively and strictly implemented, especially at the home setting.

## Introduction

Severe acute respiratory syndrome coronavirus (SARS CoV-2) is a highly transmissible coronavirus that emerged in December 2019 in Wuhan, China causing a devastating disease worldwide named coronavirus disease (COVID-19)^1–3^. It has spread overwhelmingly beyond its predecessors SARS and MERS, in terms of infected figures worldwide and geographical coverage^1^. SARS CoV-2 is transmitted through respiratory droplets emitted by coughing, sneezing or talking, allowing the virus to enter the respiratory system of the susceptible agent^4^. Moreover, the virus can cause infection through contaminated hands that make contact with mucous membranes of the eyes, mouth, and nose.

As of July 27, 2021, globally, the number of confirmed cases exceeds 194 million and more than 4 million people have died from COVID-19. On the other hand, as of July 26, 2021, a total of 3,696,135,440 vaccine doses have been administered in the population^5^. The highest number of cases confirmed is found in the Americas (76,182,529), followed by Europe (59,312,787), South East Asia (37,755,175), Eastern Mediterranean (12,255,849), Africa (4,813,735) and Western Pacific (4,287,201). The 12 countries with the highest number of confirmed cases of COVID-19 to this date are: United States of America (34,256,255); India (31,440,951); Brazil (19,688, 663), Russian Federation (6,172,812); France (5,876,269); United Kingdom (5,722,302); Turkey (5,618,417); Argentina (4,846,615); Colombia (4,727,846); Spain (4,342,054); Italy (4,320,530) and Germany (3,758,401).

In early 2020, the development of vaccines against SARS-CoV-2 began. Several were authorized for emergency use in different countries by the end of 2020 and the beginning of 2021^6^, such as the Pfizer-BioNTech BNT162b2 vaccine^7^, the Oxford-AstraZeneca vaccine^8^, the Moderna vaccine^9^ or the SinoVac-CoronaVac vaccine^10–12^. Even with vaccination campaigns in roll-out, non-pharmaceutical interventions such as keeping social distance, wearing face-masks, and avoiding large gatherings remain mostly recommended and are essential to control the burden of the pandemic^13^, especially considering the thread of potentially immune-escaping variants^14^. Additional to social distancing measures, a way to prevent local outbreaks in workplaces, schools, and other locations is implementing effective testing, contact tracing, and quarantine procedures^15–17^.

During the current pandemic, it has become more evident that the spread of COVID-19 primarily depends on factors that strongly relate to human behavior, individual lifestyles, and ways in which people socialize^18,19^. Considering the relaxation of self-care rules plays an important role when modeling and forecasting the spread of COVID-19^20^. Hence, while the world is experiencing a global pandemic, the dynamics of social behavior and human contacts have reached a new level of importance. Close contacts are responsible for the transmission of a wide range of diseases^18^, as well as differences in contacting others become crucial to understand disease dynamics. In fact, social activities and cultural networks play a fundamental role when analyzing contact behavior and contact dynamics that are sensitive to people’s daily activities^21^. For instance, individuals’ type of social encounters differ when going shopping or spending time at work. In particular, a European large-scale cross-sectional pre-pandemic survey revealed detailed contact patterns^22^, showing that the number of close contacts per person per day significantly vary depending on the location, including home, work, school, etc., and also differ per country, showing for instance, that the contact rate of a person from Italy almost triples the rate from a German. Additionally, a study conducted in the United States that aimed to determine the prevalence and treatment of latent Tuberculosis, revealed the importance of accounting for the number of contacts made at different workplaces daily^23^. This study showed that a good percentage of workplaces involved around 20 or more contacts per day; the largest number of reported contacts was 124 within a bank. A further relevant issue is a difference between countries regarding teachers/pupils ratios that affect the contact dynamics in educational facilities. The average U.S. pupil/teacher ratio in public schools is approximately 15.3^24^. Nevertheless, the pupil/teacher ratio of primary and secondary education in developing countries such as the Middle East/North Africa, Sub-Saharan, Latin America, and others Caribbean in conjunction with East and South Asian countries range from 21 to 35 on average^25^. According to UNESCO, the pupil-teacher ratio (PTR), computed as the total number of pupils in a particular school divided by the total number of teachers, was calculated for public primary schools in Nigeria, revealing a worse scenario with PTRs greater than 50^26^. Those contact differences between individuals is a crucial element for understanding how diseases are transmitted^18^.

Intervention measures that reduce social distancing can reduce effective contacts for disease transmission at any location^27^. In fact, it has been observed that the probability of infection between susceptible and infectious decreases with distance^28^. In every population, in the absence of disease, there is a natural social distance that is culture and location-dependent (home, work, school, etc.). Theories on human behaviors state that environmental factors, psycho-social aspects, and cultural backgrounds influence those distances that individuals maintain to each other in their daily lives^29–33^. However, social distances between individuals change from their natural ones in the presence of a pandemic situation^34^. Under such circumstances, individuals change their awareness by controlling their social distancing and contacting behavior, either spontaneously for protecting themselves or complying to government-mandatory interventions^34–36^.

Social science support in the field of human behavior is needed to align with epidemiological recommendations and to better understand the evolution of COVID-19. In this respect, different perspectives have been presented by the authors in^37^ for identifying insights regarding social and behavioral approaches during the current pandemic. Such findings show that a strong fear might change the behavior of the people, only if they feel a sense of efficacy in the measure taken. This study revealed a bias optimism among certain people: some of them belief that bad things are unlikely to happen to them which on one hand could avoid negative thoughts, but on the other hand could underestimate the impact of the disease. Consequently, depending of their perception about health risk, people might return to “normal life” by relaxing their protective measures from increased social distancing to their natural distancing and contacting behavior, providing favorable conditions for disease transmission^38^. Furthermore, scientific studies regarding dispositional resistance to change have been studied in different parts of the world^39,40^ revealing cultural differences amongst groups of people in terms of strong or weak resistance to change.

Another important issue that needs to be clarified is how people across the world spend their time and where they gather. Evidences suggest that time spent at each location (e.g. work, school, leisure activities, etc.) also depend on cultural practices^41,42^. A multicultural study conducted by the University of Oxford, revealed information referred to paid work and study hours distributed per week and weekends^42^ using a big data set that included information from: Belgium, Finland, Germany, Italy, Norway, Spain, Sweden, Bulgaria, Estonia, Latvia, Lithuania, Poland, Slovenia, Japan, Brazil and United States. In addition the *American Time Use Survey (ATUS)*^41^ determined the amount of time individuals in the United States spend on various activities, such as: home-activities, work, educational and leisure activities. From those studies, it can be concluded that individuals from Asian countries, Baltic states and the United States are the ones who spend greater time at work compared to other countries. Also, a report made by Data Quality Experian^43^ detailed the scheduled time Americans spend throughout the day (24 h) via an infographic picture. Based on this information, it can be said, for instance, that the largest number of people in the United States go to sleep at 3:00 a.m.; between 6:00 to 8:00 a.m., there is a sharp rise of people working or performing educational activities. Another digital source of the database is Statista^44^, reporting that a US citizen’s average daily time shopping is around 29 min. Finally, a recent platform for local mobility provides information about people’s mobility, clustered by types of activities for different communities around the world during COVID-19 times^45^. This mobility information shows a percentual reduction/increment of mobility for each activity type compared to a baseline pre-pandemic mobility index.

Current literature evidences many mathematical models that explain, characterize, and project the evolution of different infectious diseases that affect human beings^46–50^. In particular, plenty of mathematical models for COVID-19 dynamics have been developed recently^51–54^. Many of the constructed models are extensions of the classical Kermack-McKendrick Susceptible-Infectious-Recovered (SIR) epidemic model^55^, considering additionally asymptomatic cases^53,56^, hospitalized individuals, quarantine strategy^56–58^ and/or disease induced deaths^53^.

The complexity in capturing infectious diseases spread has lead us to incorporate human interactions and reactions within epidemic models^18^. Most classical models have assumed that all parameters related to human behavior such as contact rates are static, omitting that individuals are likely to change their behavior once they are exposed to the risk of acquiring a potentially deadly or dangerous disease^38^. In particular, it has been previously suggested to incorporate social-behavioral factors within mathematical disease models to understand better how personal interactions in a society affect disease transmissions, such as physical contact, social distancing behavior, and social mapping of activities^38^. As a result, a wide range of models that extend the classical approach include dynamic transmission rates, which often depend on the evolution of infectious individuals and human behavioral variables^59–68^. In particular, recent scientific evidence has acknowledged the use of incorporating changing human behavior within the mechanisms of transmission of the novel SARS-CoV-2^51,53,54,57^. Some other models account for human behavior by dividing the population into different risk groups to consider disease awareness, risk, or fear levels, among others^69–78^. For instance, in^77^, the authors show a deterministic mathematical model of differential equations and an agent-based model that incorporates the dynamics of “fear” to acquire a disease, showing that when fear subsides, it can produce multiple new waves of infection. In^78^, the authors include behavioral change by studying disease dynamics, including active and less active groups of people, showing that disease dynamics are sensitive to the transfer rate between both groups. Another study^79^ incorporates social behavior in a compartmental form by presenting the competing dynamics between a resident pathogenic strain and a mutant strain with higher virulence using imitation dynamics^80^. In addition, a mathematical model, which quantifies the epidemiological impact of the size of groups of individuals, who do or do not follow responsible behavior, can be found in^75^. Moreover, in the context of COVID-19, an agent-based model was presented by the authors in^81^, which study non-pharmaceutical intervention scenarios by reducing contact rates in specific settings/locations, but without considering adaptive behavior due to disease presence. In^82^, the authors describe the importance of non-pharmaceutical interventions, particularly the use of face-masks, based on a model of ordinary differential equations, modeling face-mask use as a proportion that lowers the probability of transmission of the entire population. Finally, a modeling study in^17^ combined a model of individual-level transmission stratified by setting, strategies of self-isolation, testing, contact tracing, and also physical distancing to ensure the effectiveness of interventions against SARS-CoV-2 . The aforementioned study showed that only self-isolation of symptomatic cases reduced the transmission rate up to 29%, whilst adding household quarantine increased the transmission reduction to 37%. This leads us to think that a stratified mathematical modeling approach aggregated by different settings such as household, work, school, and others, might provide a more accurate scenario for disease control.

We complement the existing literature by extending a compartmental model of ordinary differential equations by considering three main locations where individuals make contacts: home (*h*), work (*w*), school (*c*), and other locations (*o*), for instance, those destined to leisure activities. At each location, we propose a disease progression according to a Susceptible-Exposed-Infectious-Recovered (SEIR) model, where infectious individuals are divided into symptomatic and asymptomatic, and those infectious individuals in conjunction with individuals that are encountered by them, become quarantined for a certain period of time. Additionally, we incorporate social distancing at each location as a dependent variable described by a differential equation. The focus of this dynamic might depend on two opposite drives: the fear that people perceive due to the point prevalence of the disease as opposed to the resistance individuals experience to change social distancing behavior. All under the premise of a population that is culturally accustomed to behave following a certain natural distance. In addition, the transmission rate of a disease depends on the proximity of contacts between one susceptible and one infectious individual^18^. Hence, in the proposed model, a transmission rate function will depend on the dynamics of the social distancing variable described before. The behavioral parameters in the present formulation will reflect how people behave daily, considering: (1) where individuals choose to go each day; (2) average time spent at the chosen location; and (3) behavioral aspects of fear and resistance to change, which affect social distancing dynamics at each location and consequently affect disease transmission.

To the best of our knowledge, no studies have been conducted using epidemiological modeling, structured by location and location-dependent social distancing dynamics, which is governed by disease perception and awareness. This study intends, first, to understand how different location-targeted restrictive measures applied overall or at different daily time-blocks affect disease dynamics; second, how a change in location-dependent social distancing affects disease dynamics; and third, how a policy measure of quarantining a person upon a close contact with an infected person might affect disease propagation at different locations.

## Methodology

### Model in the absence of disease

In this subsection we present the model in the absence of disease. Without considering demographic factors, we assume a population of constant size *N*. We stratify the population according to different settings: home (*h*), work (*w*), school (*c*) and other (*o*), such that $N_h$, $N_w$, $N_c$ and $N_o$ are respectively the number of individuals at each setting, i.e. $N=N_{h}+\sum _{x \in X}N_{x}=\sum _{y\in Y}N_{y}$ is the total population size, where $X=\{w,c,o\}$ and $Y=\{h,w,c,o\}$.

We assume that individuals that are at home leave home at a rate $\delta$ in order to visit places in *X*, distributed such that a proportion $\alpha _x \ge 0$ goes from home to location $x\in \{w,c,o\}$ and $\sum \alpha _{x}=1$. Once an individual visits location $x\in \{w,c,o\}$, the average time spent at that location is $\sigma _x$, and hence, the exit rate from location *x* back to home is $1/\sigma _x$. It is important to notice that the base location is home, i.e. there are no transitions between locations $x\in \{w,c,o\}$. The mobility dynamic can be represented by the following system of differential equations:

1 $$\begin{aligned} \left\{ \begin{array}{rcl} {\dot{N}}_{h} &{} = &{} -\delta (\sum \alpha _{x}) N_{h}+\sum \frac{1}{\sigma _{x}}N_{x}, \\ {\dot{N}}_{x} &{} = &{} +\delta \alpha _{x} N_{h} - \frac{1}{\sigma _{x}} N_{x},\quad x\in X, \quad \sum \alpha _{x}=1. \end{array} \right. \end{aligned}$$

Considering that the time-unit is days, it is reasonable to assume that the rate $\delta$ and the proportions of how the location settings are distributed, $\alpha _x, x\in$ $\{w,c,o\}$, differ according to the day of the week (e.g. weekday or weekend) and also according to dynamics that occur during a day (e.g. daytime or night). In that sense, we divide each day in six consecutive time-blocks of equal duration $H=\{1{st},\,2{nd},\,3{rd},\,4{th},\,5{th},\,6{th}\}$ and the week in 7 days a week $W=\{Mon,\,Tue,\,Wed,Thu,\,Fri,\,Sat,\,Sun\}$, such that the notation

$$\begin{aligned} \delta (i,j) \quad \alpha _{x}(i,j) \quad \text{ and } \quad \sigma _{x}(i,j), \quad \text{ with }\quad (i,j) \in H\times W, \end{aligned}$$

determines the value of each parameter in each time block according to *i*, and each day of the week according to *j*.

**Figure 1:**
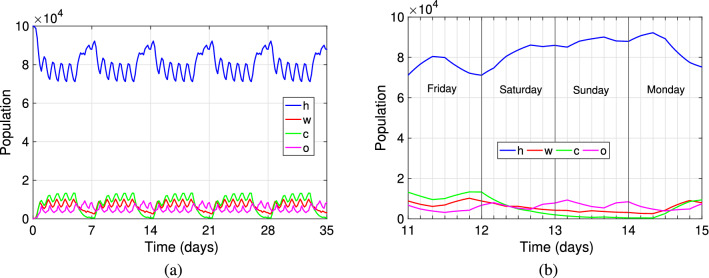
Daily distribution of the population in the spatial compartments of **h**-home, **w**-work, **c**-educational facilities, and **o**-other activities. In (**a**) the dynamics for five consecutive weeks. In (**b**) the dynamics during 4 days from Friday to Monday to differentiate workday and weekend, depicting within each day six time-blocks. The parameters involved are detailed in Table 1 with a total population size $N=100{,}000$.

Figure 1 shows the mobility dynamics of the population in the absence of disease, at the locations home (blue), work (red), school (c) and other locations (purple) with time. Figure 1a shows the mobility for five consecutive weeks, starting on a Monday, with everybody at the home setting. We can observe a weekly periodic pattern, where the home location is the most visited. We see during the weekend an increase in home visits and a decrease in school and work locations, as expected. The zick-zack pattern represents the six time blocks per day, at which mobility dynamics change. Figure 1b depicts the dynamics between a Friday and the coming Monday, for each of the six daily time-blocks at each setting. The figure shows clearly, for instance, the decrease in work and school activities starting Saturday and lasting till Monday morning, while leisure and home activities increase during that time. Table 1 shows the parameter values at each time-block and day of the week used for Fig. 1.

**Table 1:** Parameter values used in Fig. 1 and obtained from the references listed in Table 4, found at the end of the “Numerical results” section.

$10^{4}\delta$ $10^{2}(\alpha _{w},\alpha _{c},\alpha _{o})$ $(\sigma _{w},\sigma _{c},\sigma _{o})$	1st 9 p.m.–1 a.m.	2nd 1 a.m.–5 a.m.	3rd 5 a.m.–9 a.m.	4th 9 a.m.–1 p.m.	5th 1 p.m. – 5 p.m.	6th 5 p.m.– 9 p.m.
$Mon \rightarrow Fri$	220	645	3585	7095	72,235	6060
	(50,30,20)	(50, 0, 50)	(45,45,10)	(40,40,20)	(40,40,20)	(10,30,60)
	(3, 4, 2)/4	(3, 4, 2)/4	(3, 4, 2)/4	(3, 4, 2)/4	(3, 4, 2)/4	(3, 4, 2)/4
$Sat \,\rightarrow \, Sun$	3000	100	1000	1000	3000	2000
	(20, 0,80)	(10, 0, 0)	(100, 0, 0)	(30,30,40)	(10, 0,90)	(20, 0,80)
	(3, 2, 3)	(3, 2, 3)	(3, 2, 3)	(3, 2, 3)	(3, 2, 3)	(3, 2, 3)

### Model in the presence of disease

Here we extend the simple mobility model from the previous subsection by including disease dynamics. In particular, we present a model for COVID-19 dynamics with quarantine, mobility and distancing behavior that differs according to location. The model splits the population into six epidemiological classes: Susceptible (*S*), Latent (*E*), Asymptomatic (*A*), Infectious (*I*), Quarantined (*Q*) and Recovered (*R*). We assume that latent individuals cannot transmit the disease, that asymptomatic do not show symptoms and that infectious individuals are symptomatic, and that asymptomatic individuals and infectious individuals are capable of transmitting the disease.

**Figure 2:**
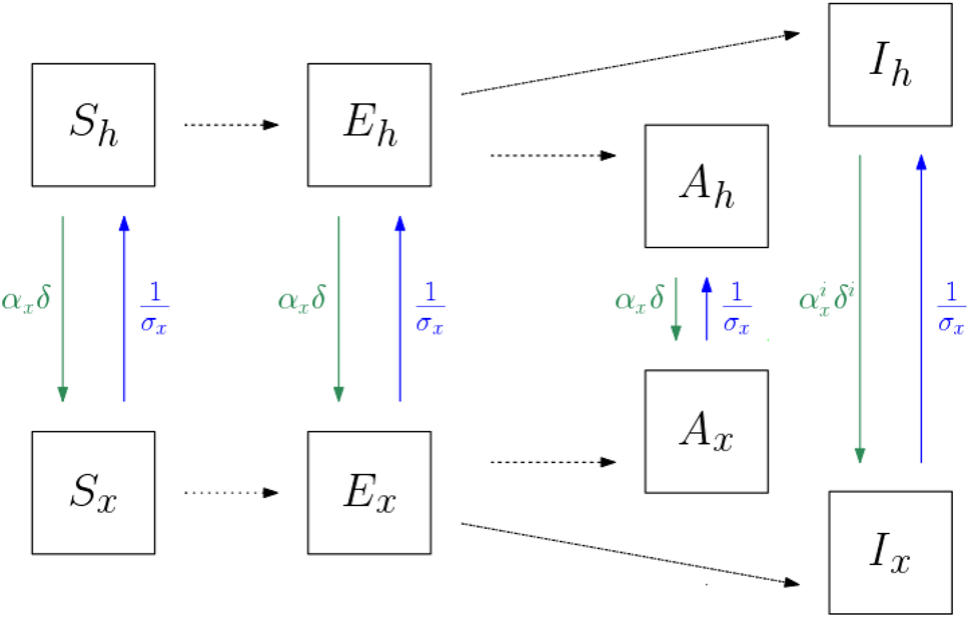
Schematics showing the flow between Susceptible (*S*), Latent (*E*), Infectious (*I*), Asymptomatic (*A*) individuals, between the home location represented by the subscript *h*, and other locations represented by the subscript *x*, where $x\in \{w,c,o\}$, such that *w* represents work, *c* educational facilities, and *o* other locations for example for leisure activities. The black dotted arrows represent transition rates that are due to disease dynamics and are described in Fig. 3. See Table 2 for the description of the parameters of this diagram.

We assume for the classes *S*, *E*, *A* and *I*, that individuals spend their time at home (*h*), work (*w*), educational facilities (*c*) and doing other leisure activities (*o*). We assume that susceptible, latent and asymptomatic individuals that are at home leave their home at a rate $\delta$, such that a proportion $\alpha _x$ goes from home to location $x\in \{w,c,o\}$. This proportion does not change according to if the person is susceptible, latent or asymptomatic, since at those stages individuals may not even realize that they are infected and hence wouldn’t change their behavior. Infectious individuals that are at home leave their home at a rate $\delta ^i<\delta$, where a proportion $\alpha _x^i$ visits location $x\in \{w,c,o\}$. Once an individual—of any of the epidemiological classes—visits location *x*, the time spent at that location is $\sigma _x$, and hence, the exit rate from location *x* is $1/\sigma _x$. Figure 2 describes the behavioral flow of individuals, between home and location *x*.

We assume that individuals change their interaction distance with others depending on where they are located. We denote that *interaction distance* by $D_y(t)$, where $y\in \{h,w,c,o\}$ represents home, work, school and others, respectively, whose dynamics is described by the equation

2 $$\begin{aligned} {\dot{D}}_{y} = -\lambda _{1}(D_{y}-D_{y}^{*}) + \lambda _{2}^{y}\sum _{y}(I_{y}+dQ_{i})/N, \end{aligned}$$

where $\lambda _1$ represents the rate of resistance to change with respect to the natural distance $D^*_y$ that people keep from each other in the absence of disease; $\lambda _2^y$ represents the per capita rate of change of the distance as a reaction to the number of infectious individuals; and *N* the total population size.

**Figure 3:**
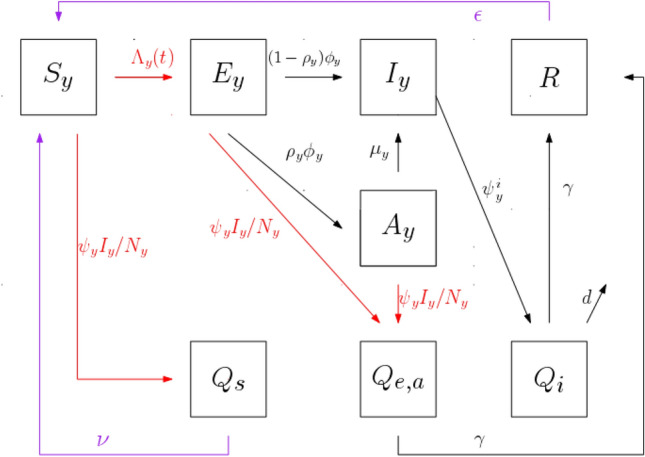
Schematics showing the flow between Susceptible ($S_y$), Latent ($E_y$), Infectious ($I_y$), Asymptomatic ($A_y$), Recovered (*R*) and Quarantined ($Q_i$, $Q_{e,a}$, $Q_s$) individuals, for each location $y\in \{h,w,c,o\}$, such that *h* represents home, *w* work, *c* educational facilities, and *o* other locations for example for leisure activities. The read arrows represent rates that depend upon a contact with infectious individuals $I_y$. The dashed-dotted purple arrow just holds when $y=h$. See Table 3 for the description of the parameters.

The diagram in Fig. 3 shows the flow between the epidemiological classes. This disease dynamics holds for any location $y\in \{h,w,c,o\}$.

Susceptible individuals can become latent when contacting an infectious or asymptomatic from the same location, according to the following force of infection

3 $$\begin{aligned} \Lambda _y(t):=\beta _a^y(D_y,\Theta _{y})\dfrac{A_y(t)}{N_y(t)} + \beta _i^y(D_y,\Theta _{y})\dfrac{I_y(t)}{N_y(t)}, \qquad y\in \{h,w,c,o\}, \end{aligned}$$

where $\beta _a^y(D_y,\Theta _{y})$ and $\beta _i^y(D_y,\Theta _{y})$ represent, respectively, the transmission rates upon a contact of a susceptible with an asymptomatic (subscript *a*) and with an infectious (subscript *i*) individual. In particular, the transmission rate at home ($y=h$) is defined as:

4 $$\begin{aligned} \beta _z^h(D_h,\Theta _{h}):=\beta _{z}^h\dfrac{D_h^*}{D_h(t)}\Theta _{h}, \qquad z\in \{a,i\}, \end{aligned}$$

where $D_h^*$ is the natural distance held at home and $\Theta _h$ a parameter that considers the reduction in transmission at home due to home-exit restrictions. More specifically, observe that the transmission rate decreases when $D_h(t)$ increases from the natural distance $D_h^*$. When $D_h(t)$ is equal to $D_h^*$, the disease gets transmitted at a base line transmission rate $\beta _z^h$, times the parameter $\Theta _h$. The latter represents the reduction in the home-transmission rate when reducing by $Red_x$ the proportions $\alpha _x^*$, $x\in \{w,c,o\}$, which are the proportions by which people leave the home (*h*) location under normal conditions. This way, $\Theta _h$ is defined as

5 $$\begin{aligned} \Theta _{h}:=(1-Red_w)\alpha _w^* + (1-Red_c)\alpha _c^* + (1-Red_o)\alpha _o^*. \end{aligned}$$

Equivalently, the transmission rates at work ($y=w$), school ($y=c$), and other locations ($y=o$) are defined as

6 $$\begin{aligned} \beta _z^y(D_y,\Theta _{y}):=\beta _{z}^y\dfrac{D_y^*}{D_y(t)}\Theta _{y}, \qquad z\in \{a,i\}, \; y\in \{w,c,o\}, \end{aligned}$$

where the respective reductions in the transmission rates at those locations, due to home-exit restrictions, are

7 $$\begin{aligned} \Theta _{w}= & {} (1-Red_w)\alpha _w^* + \alpha _c^* + \alpha _o^* \end{aligned}$$

8 $$\begin{aligned} \Theta _{c}= & {} \alpha _w^* + (1-Red_c)\alpha _c^* + \alpha _o^* \end{aligned}$$

9 $$\begin{aligned} \Theta _{o}= & {} \alpha _w^* + \alpha _c^* + (1-Red_o)\alpha _o^*. \end{aligned}$$

From the definitions of the parameters $\Theta _y$, $y\in \{h,w,c,o\}$, observe that we assume that the home-transmission-rate gets reduced whenever people choose not to go to any of the other locations; while the transmission-rate at work, school, or other locations, get reduced only when people choose not to go to that specific location, i.e. work, school, other locations, respectively.

Individuals exit the latent class at a rate $\phi _y$, of which a proportion $\rho _y$ becomes asymptomatic and a proportion $1-\rho _y$ becomes infectious (symptomatic). Infectious individuals become quarantined at a rate $\psi _y^i$, entering the $Q_i$ class, and then die from the disease at a rate *d* or recover at a rate $\gamma$. Asymptomatic individuals become infectious at a rate $\mu _y$. Asymptomatic and latent individuals become quarantined upon a close contact with an infectious person, entering the $Q_{e,a}$ class, both at a rate $\psi _yI_y/N_y$, and then recover at a rate $\gamma$. Susceptible individuals become quarantined also at a rate $\psi _y I_y/N_y$ upon a close contact with an infectious person, entering the class $Q_s$, and then return home at a rate $\nu$. Recovered individuals may loose immunity and return home as susceptible individuals at a rate $\epsilon$.

Using the schematics in Figs. 2 and 3 and the above description, we obtain the following system of ordinary differential equations governing the dynamics of the disease:

10 $$\begin{aligned} \left\{ \begin{array}{l} \begin{aligned} {\dot{S}}_{h} &{}= -\delta (\sum _x \alpha _x) S_{h} +\sum _{x}^{}\frac{1}{\sigma _{x}}S_{x} -S_{h}\Lambda _{h} - \psi _{h}S_{h}I_{h}/N_{h} + \nu Q_{s} + \epsilon R\\ {\dot{S}}_{x} &{}= \alpha _{x}\delta S_{h} - \frac{1}{\sigma _{x}}S_{x} -S_{x}\Lambda _{x} -\psi _{x}S_{x}I_{x}/N_{x}\\ {\dot{E}}_{h} &{}= -(\delta \sum _x \alpha _x + \phi _{h}) E_{h} +\sum _{x}^{}\frac{1}{\sigma _{x}}E_{x} +S_{h}\Lambda _{h} - \psi _{h}E_{h}I_{h}/N_{h}\\ {\dot{E}}_{x} &{}= \alpha _{x}\delta E_{h} -\frac{1}{\sigma _{x}}E_{x} +S_{x}\Lambda _{x}-\phi _{x}E_{x} - \psi _{x}E_{x}I_{x}/N_{x}\\ {\dot{A}}_{h} &{}=-(\delta \sum _x \alpha _x + \mu _{h})A_{h} +\sum _{x}^{}\frac{1}{\sigma _{x}}A_{x} + \rho _{h}\phi _{h}E_{h} -\psi _{h}A_{h}I_{h}/N_{h}\\ {\dot{A}}_{x} &{}= \alpha _{x}\delta A_{h} -\frac{1}{\sigma _{x}}A_{x} + \rho _{x}\phi _{x}E_{x} - \mu _{x}A_{x}-\psi _{x}A_{x}I_{x}/N_{x}\\ {\dot{I}}_{h} &{}=-(\delta ^{i} \sum _x \alpha _x^i + \psi _{h}^{i})I_{h} +\sum _{x}^{}\frac{1}{\sigma _{x}}I_{x} + (1-\rho _{h})\phi _{h}E_{h} + \mu _{h}A_{h}\\ {\dot{I}}_{x} &{}= \alpha _{x}^{i}\delta ^{i} I_{h} -\frac{1}{\sigma _{x}}I_{x} + (1-\rho _{x})\phi _{x}E_{x} + \mu _{x}A_{x} - \psi ^{i}_{x} I_{x}\\ {\dot{Q}}_{s} &{}= \sum _{y}^{}\psi _{y}S_{y}I_{y}/N_{y} - \nu Q_{s}\\ {\dot{Q}}_{e,a} &{}= \sum _{y}^{} \psi _{y}(E_{y} + A_{y}) I_{y}/N_{y} - \gamma Q_{e,a}\\ {\dot{Q}}_{i} &{}= \sum _{y}^{} \psi ^{i}_{y}I_{y} - (d+\gamma )Q_{i}\\ {\dot{R}} &{}= \gamma (Q_{e,a} + Q_{i}) - \epsilon R\\ {\dot{D}}_{y} &{}= -\lambda _{1}(D_{y}-D_{y}^{*}) + \lambda _{2}^{y}\left( \sum _{y}^{}I_{y} + d Q_{i}\right) /N \\ {\dot{M}} &{}=dQ_{i} \end{aligned} \end{array} \right. \end{aligned}$$

where $$x\in \{w,c,o\}$$, $$y\in \{h,w,c,o\}$$, $$N_{y}=S_{y}+E_{y}+A_{y}+I_{y}$$, and $$N=\displaystyle \sum _{y}N_y.$$

**Table 2:** Description of parameters and parameter values related to behavior.

Parameters	Description	Units	Baseline^a^	Ref.
$\delta$	Home exit rate for all, except for infectious individuals	$d^{-1}$	24/13.34	^41^
$\delta ^i$	Home exit rate for infectious individuals	$d^{-1}$	$0.5*\delta$	Author chosen
$\alpha _w$ ($\alpha _w^{i}$)	Proportion of non-infectious (infectious) individuals going from home to work	Unitless	3.57/10.66	^41^
$\alpha _c$ ($\alpha _c^{i}$)	Proportion of non-infectious (infectious) individuals going from home to educational facility	Unitless	0.46/10.66	^41^
$\alpha _o$ ($\alpha _o^{i}$)	Proportion of non-infectious (infectious) individuals going from home to perform other (e.g. leisure) activities	Unitless	6.63/10.66	^41^
$\sigma _w$	Average time spent at work	*d*	3.57/24	^41^
$\sigma _c$	Average time spent at educational facilities	*d*	0.46/24	^41^
$\sigma _o$	Average time spent at other (leisure) locations	*d*	6.63/24	^41^
$\lambda _1$	Rate of resistance to behavioral change	$d^{-1}$	$\left[ 0,1\right]$	^39^
$\lambda _2^y$	Per capita rate of change of distancing behavior when at location *y*	$m\,d^{-1}$	$\left[ 0,1\right]$	Author chosen
$D^*_y$	Scaling distance relative to location *y*	*m*	$\left[ 0.5,2\right]$	^33^

d: = day, m: = meters.

^a^Average numerical value, the breakdown by blocks is listed in Table 4, found at the end of the “Numerical results” section.

**Table 3:** Description of epidemiological parameters and parameter values.

Parameters	Description	Units	Value	Baseline	Ref.
$\psi _y$	Rate of transition to quarantine upon a close contact at location *y*, of *S*, *E* or *A* with *I*	$d^{-1}$	$\left[ 1.88*0.35\,,\,3.08*0.75\right]$	^a^	^22^
$\beta _a^y$	Transmission rate of asymptomatic individual at location *x*	$d^{-1}$	$\psi _y*0.3$	$\psi _y*0.3$	Author chosen
$\beta _i^y$	Transmission rate of infectious individual at location *x*	$d^{-1}$	$\beta _{a}^{y}*0.5$	$\beta _{a}^{y}*0.5$	Author chosen
${\psi _y}^{i}$	Rate of transition to quarantine of *I* individuals at location *y*	$d^{-1}$	$\left[ 1/3,1\right]$	1/5	^83^
$\nu$	Quarantine recovery rate	$d^{-1}$	1/14	1/14	^83^
$\phi _y$	Exit rate from latent to asymptomatic and infectious	$d^{-1}$	$\left[ 1/7,1/3\right]$	1/5	Author chosen
$\rho _y$	Proportion of latent that transit to asymptomatic	Unitless	$\left[ 0.2,0.6\right]$	0.3	^83^
$\mu _y$	Transition rate from asymptomatic to infectious	$d^{-1}$	$\{0\}\cup \left[ 1/11,1\right]$	1/7	Author chosen
*d*	Disease induced death rate	$d^{-1}$	$\left[ 0.01,0.1\right]$	0.01	^83^
$\gamma$	Recovery rate	$d^{-1}$	1/14	1/14	^83^

^a^Average numerical value, the breakdown by blocks is listed in Table 4, found at the end of the “Numerical results” section.

d: = day, $$1/\phi _{y}+1/\mu _{y}+1/\psi _{y}^{i}\le 14$$ with $$y\in \{h,w,c,o\}$$, $$x\in \{w,c,o\}$$.

## Numerical results

We present in this section simulations that describe the effect on the number of active cases of different restrictive measures implemented at different locations and time-blocks. We also describe results on how increasing the natural distancing behavior in the home setting or improving close-contacts tracing at different locations affect disease dynamics. The code for these simulations is available in^84^.

### Location-targeted restrictive measures and its effect on the number of active cases

**Figure 4:**
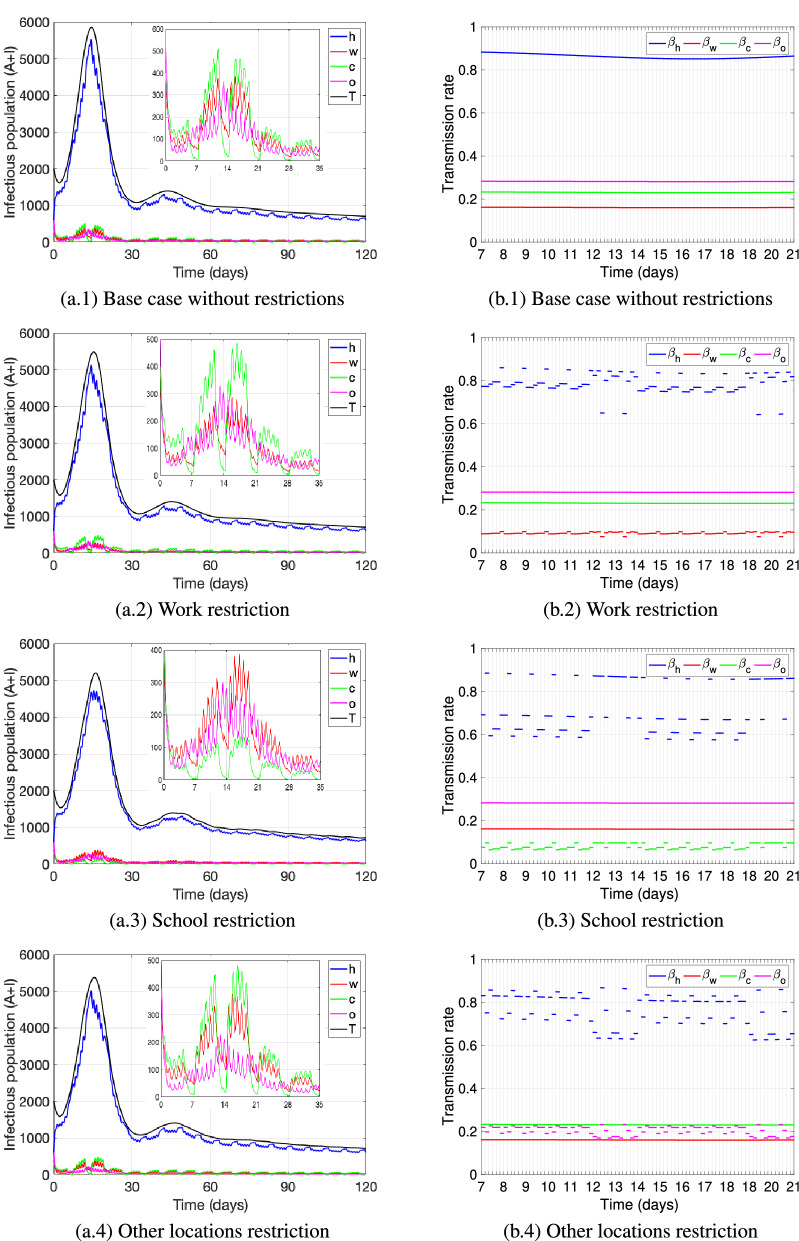
The figures (**a.i**), i = 1–4 depict the number of active cases ($A(t)+I(t)$)—the infectious population—at each location: home (*h*, blue), work (*w*, red), school (*c*, green) and other (*o*, pink); as well as the total number of active cases (*T*, black). The curves are pictured for a base case without restrictions and under one restrictions at a time to attend a specific location: (**a.1**) base case, (**a.2**) work, (**a.3**) school, (**a.4**) other. For each case, the figures (**b.i**), i = 1–4 show the respective transmission rates at each location, pictured for time blocks within each day, from day 7 to day 21. The restrictions are obtained by reducing the parameters $\alpha _x$ and $\alpha _x^i$, $x\in \{w,c,o\}$, by: $25\%$ for work, $73\%$ for school, $30\%$ for other activities. The parameter values used are as in Table 4.

**Figure 5:**
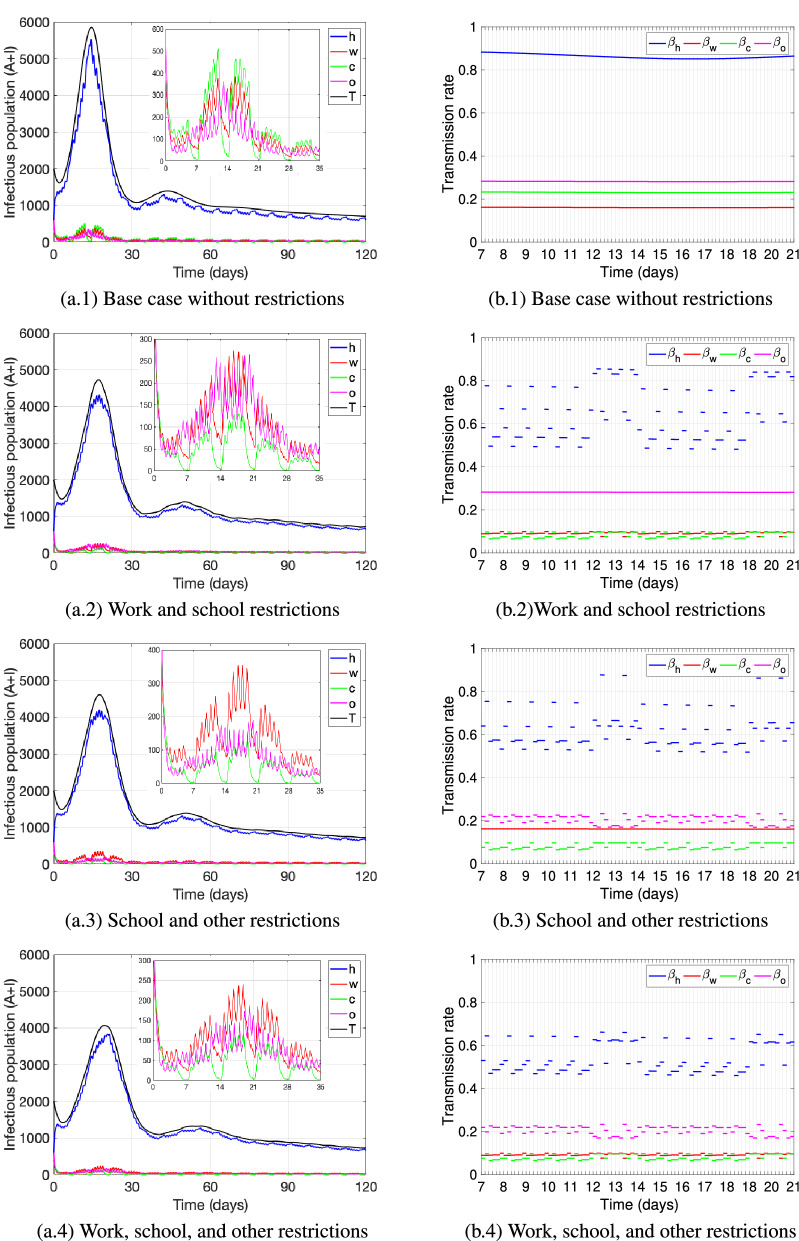
The figures (**a.i**), i = 1–4 depict the number of active cases ($A(t)+I(t)$)- the infectious population- at each location: home (*h*, blue), work (*w*, red), school (*c*, green) and other (*o*, pink); as well as the total number of active cases (*T*, black). The curves are pictured for a base case without restrictions, and under several restrictions to attend locations applied at once: (**a.1**) base case, (**a.2**) work and school, (**a.3**) school and other, (**a.4**) work, school and other. For each case, the figures (**b.i**), i=$1-4$ show the respective transmission rates at each location, pictured for time blocks within each day, from day 7 to day 21. The restrictions are obtained by reducing the parameters $\alpha _x$ and $\alpha _x^i$, $x\in \{w,c,o\}$, by: $25\%$ for work, $73\%$ for school, $30\%$ for other activities. The parameter values used are as in Table 4.

Figures 4 and 5 show the dynamics of the number of active cases $(A(t)+I(t))$ (see subfigures (a.i), i $=1-4$) and the transmission rate (see subfigures (b.i), i $=1-4$) at each of the locations: home (*h*, blue), work (*w*, red), school (*c*, green), other (*o*, pink), and for all locations combined (*T*, black); and under different restrictive measures for attending specific locations. To restrict the attendance to a location (work, school, other), we reduced the proportion of individuals going from home to that location. In the model, that proportion is represented by $\alpha _x$ for non-infectious individuals at home and $\alpha _x^i$ for infectious individuals at home, $x\in \{w,c,o\}$. We study in this section the effect on the number of active cases of the following restrictive measures: (1) reducing the proportion of individuals going from home to work by $25\%$, capturing the percentage of jobs that can be performed from home that varies within the interval $20-34\%$ from country to country^85^, (2) reducing the proportion of individuals going from home to school by $73\%$, representing a scenario of closing school activities for children older than 4 years of age, who represent $73\%$ of the child population in the United States^86^; (3) reducing the proportion of individuals going from home to other locations by $30\%$, motivated by data showing that a $15{-}30\%$ growth in consumers in the United States has been observed, who purchase online for most categories post COVID-19 as compared to before^87^.

Figure 4a.1,b.1 show the base case scenario without any restrictive measures, while Fig. 4a.2–a.4 and b.2–b.4 show the dynamics when restricting the attendance to work, school, and other locations respectively, one at a time. We observe that neither of the single restrictions pictured produce a significant reduction in the total number of active cases as compared to the base case (see black curve in Fig. 4a.1–a.4). We observe that the first peak of the active cases at home shifts slightly to the left when work and other locations attendance is restricted (Fig. 4a.2 and a.4), while it slightly flattens out when school attendance is restricted (Fig. 4a.3), being this restriction the one reducing the number of active cases the most. Additionally, we can observe that the number of active cases at school decrease when school attendance is restricted and becomes the location with the least number of active cases (Fig. 4a.3), while otherwise it can be seen that school is the location (excluding the home location) with the largest number of active cases during weekdays (Fig. 4a.1,a.2,a.4). Also, the number of active cases at other locations become as well the least (compared to work and school) when attendance to other locations is restricted (Fig. 4a.4). On the other hand, when work attendance is restricted, during weekdays the number of active cases at work still remains above the number at other locations (Fig. 4a.2).

We can observe form Fig. 4b.1–b.4 how the transmission rate at home is impacted by the restrictions at different locations that affect the transmission rate at those locations as well. The figures picture the value of the transmission rate at each location for different time blocks within each day from day 7 to 21 (which is the time frame of the first peak). We identify the school attendance restriction (Fig. 4b.3) to be the measure that mostly reduces the transmission rate at home (blue), especially around midday during weekdays, but transmission at home remains mostly high during the weekend (day 13 and 14, 20 and 21). Additionally, we can see that restricting work attendance reduces the transmission rate at work overall (Fig. 4b.2), but does not produce much variation in transmission between time-blocks at work. Nevertheless, the measure reduces the transmission rate at home, especially during the day on workdays. Finally, reducing leisure activities (other) produce a similar effect (Fig. 4b.4), and especially generates a reduction in the transmission rate at home and at other locations at specific times during the weekend.

Figure 5a.1 and b.1 show the base case scenario without any restrictive measures, while Fig. 5a.2–a.4 and b.2–b.4 show the dynamics when restricting the attendance to more than one location at once. For all scenarios pictured we reduced school attendance since it was the single measure that in Fig. 4 showed the largest impact on reducing active cases. As expected, we verify that the more restrictions are implemented the more reduction in the number of active cases can be seen. We observe that when school and one more restriction—either to attend work or attend other activities—are in place (Fig. 5a.2 and a.3), the number of active cases at home at the first peak slightly flattens out and reduces significantly as compared to the base case. The peak of the home cases reduces even more and slightly shifts to the right when attendance to work, school and other activities are restricted (Fig. 5a.4). In all of the combined scenarios of restrictive measures (Fig. 5a.2–a.4), the number of active cases at school is the least as compared to the cases at home, work or other activities. On the other hand, in any restrictive case, including the single location restrictions from Fig. 4, the number of active cases during weekends at other locations exceeds the number of cases at work, while during weekdays it is the opposite, though the latter is least pronounced when work and school attendance is reduced (Fig. 5a.2). Our results reflect the natural behavior of individuals to attend more leisure activities during the weekend and hence it is at these other locations where the majority of active cases are located when leaving the home setting. On the other hand, during weekdays and outside the home setting, without any restrictions (Fig. 5a.1) or only when restricting either work or other locations (Fig. 4a.2 and a.4), it is the school location where most of the active cases are located, whereas when applying combined restrictive measures (Fig. 5a.2–a.4) or just restricting school attendance (Fig. 4a.3) it is the work location where active cases prevail the most.

All measures pictured reduce the transmission rate at the restricted location and also have an effect on the transmission rate at home (Fig. 5b.1–b.4). The most dispersal of the transmission rate at home at different time-blocks per day is observed when restricting work and school attendance (Fig. 5b.2), reducing the home transmission rate during weekdays significantly, but in general maintaining it high during weekends. The latter is also observed for certain weekend time-blocks, when restricting attendance to school and other locations (Fig. 5b.3) and when restricting attendance to all three: work, school and other (Fig. 5b.4).

### Time-blocks and location targeted restrictive measures and its effect on the number of active cases

**Figure 6:**
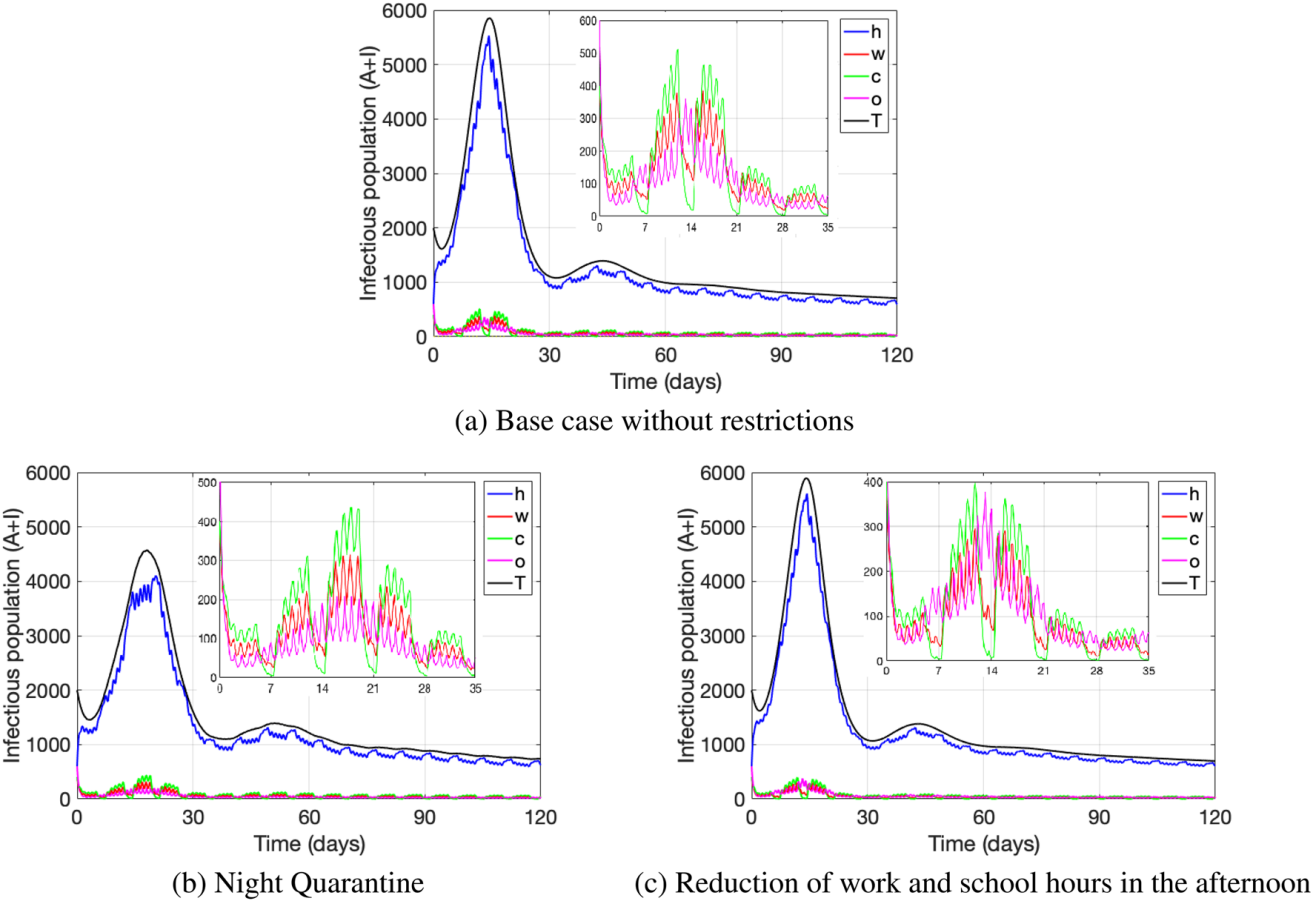
The figure depicts in each subplot the number of active cases ($A(t)+I(t)$)—the infectious population—at each location: home (*h*, blue), work (*w*, red), school (*c*, green) and other (*o*, pink); as well as the total number of active cases (*T*, black). The subplots show the curves under: (**a**) the base-case without restrictions; (**b**) a strict night-quarantine, obtained by reducing the parameters $\alpha _x$ and $\alpha _x^i$, $x\in \{w,c,o\}$ to zero for the time-blocks between 9:00 p.m. and 5:00 p.m.; (**c**) a restrictive measure reducing the time spent at work and school (represented by the parameters $\sigma _w$ and $\sigma _c$) in the afternoon by half during the time-blocks from 1:00 p.m. and 5:00 p.m. and from 5:00 p.m. to 9:00 p.m. The parameter values used are as in Table 4.

Figure 6 depicts the dynamics of the number of active cases $(A(t)+I(t))$ at each of the locations: home (*h*, blue), work (*w*, red), school (*c*, green), other (*o*, pink), and for all locations combined (*T*, black) under restrictive measures that target specific time-blocks during the day. Figure 6a shows the base case without restrictions. Figure 6b shows a scenario of night quarantine, where the proportions of individuals leaving the home setting—represented by $\alpha _x$ and $\alpha _x^i$, $x\in \{w,c,o\}$—are reduced to zero for the time-blocks between 9:00 p.m. and 5:00 p.m. On the other hand, Fig. 6c represents the restrictive measure of reducing work and school hours in the afternoon, reducing by half the times spent at work and school (represented by the parameters $\sigma _w$ and $\sigma _c$) during the time-blocks from 1:00 p.m. and 5:00 p.m. and from 5:00 p.m. to 9:00 p.m.

We can observe from Fig. 6b that if night quarantine is fully met, that is, nobody leaves the home setting, the first peak of active cases decreases at all locations and slightly flattens out in the home setting. On the other hand, no decline in the total number of active cases is observed in Fig. 6c when reducing work and school hours in the afternoon by half. On the contrary, there is a slight increase in the size of the first peak of the total number of active cases as compared to the base case without restrictions (5894 cases vs 5852, respectively). Nevertheless, a reduction in infectious cases is observed at work and school, which are probably shifted to the home setting due to individuals staying in the home setting instead of going to work or school and due to the aforementioned observed increase of total active cases in the population.

### Natural-distancing in the home setting $D_h^*$ and its effect on the number of active cases

**Figure 7:**
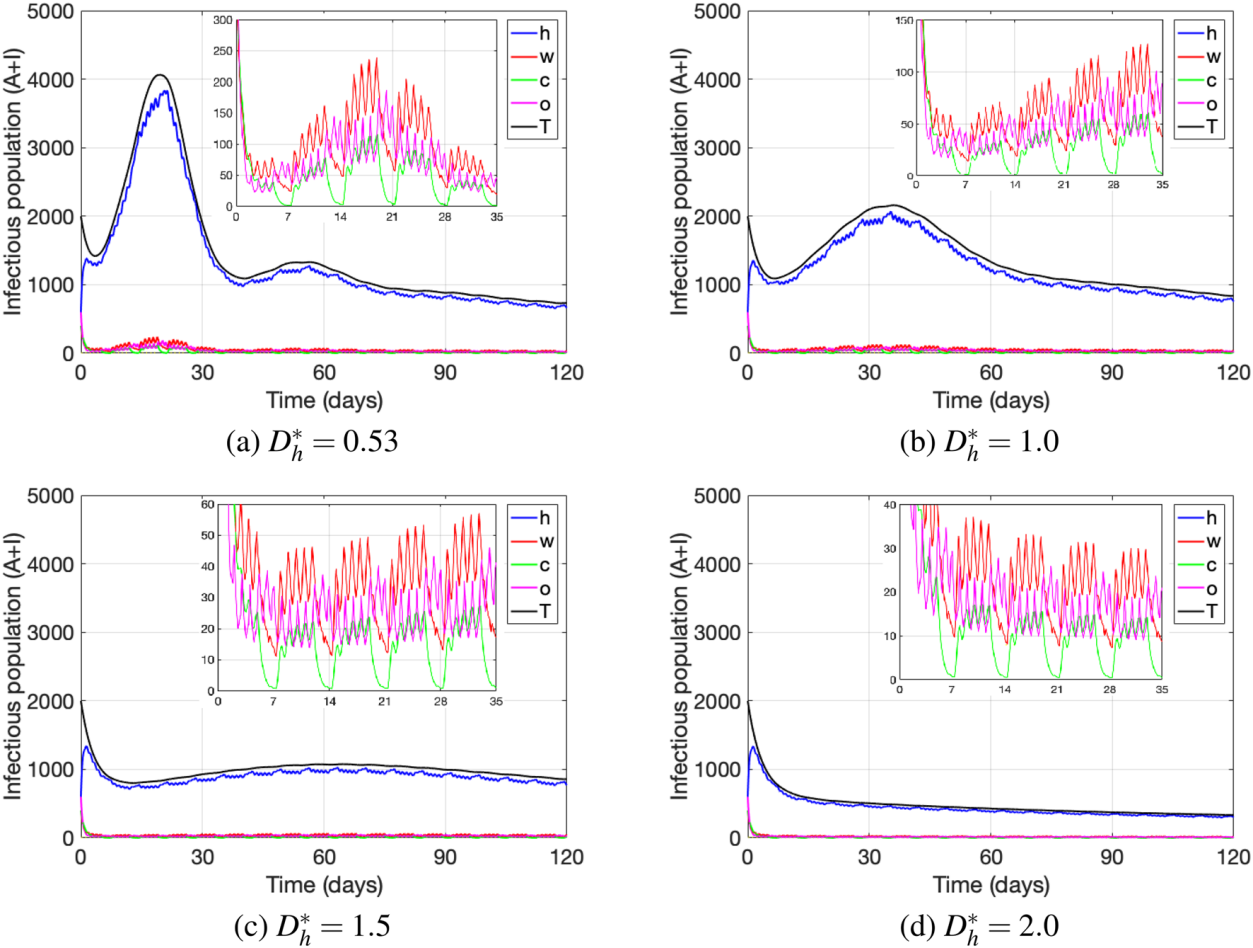
The sub-figures show the dynamics of active cases for different natural distances at home, under the measure where simultaneously going to work, school, and other locations is restricted. The parameter values used are as in Table 4.

Figure 7 shows how different natural distance values $D_h^*$ at home affect disease dynamics under the measure that restricts mobility from home to work, school and other locations. When comparing Fig. 7a–d we observe that, the greater the natural distance $D_h^*$ at home is, the later occurs the first peak of total cases and the smaller is its size, such that large $D_h^*$ values eventually flatten out the infection curve of total cases (see Fig. 7d). Hence, increasing the natural distance at home may reduce the number of active cases significantly. In particular, Fig. 7 shows that the average physical natural distance at home $D_h^*$ represents a serious threat for disease transmission when kept low, and on the contrary, it positively impacts disease reduction when kept high (see Fig. 7a,b vs  7c,d). Finally, changing the natural distance at home does not affect the dynamics between locations, being work the location with most of the cases during weekdays and being other activities locations the ones with the highest infectious population during weekends. Nevertheless, reducing natural distancing in the home setting also reduces the number of infectious cases at each of the other locations. Therefore, the inclusion of a responsible behavior at home is essential to reduce the infection curve as a whole.

### Close-contacts tracing and its effect on the number of active cases

**Figure 8:**
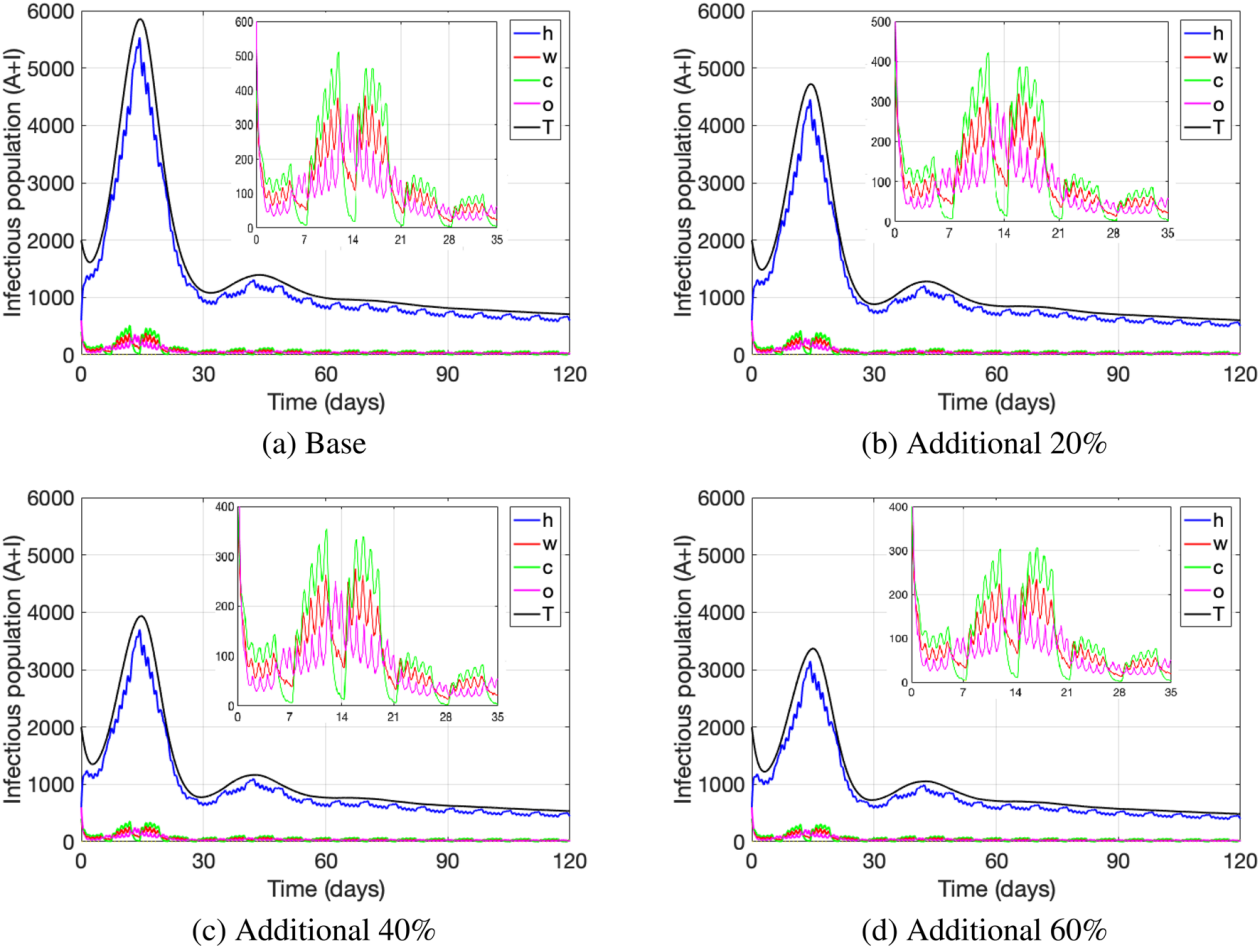
Addition (in percentage) to the transition rate leading to quarantine upon a close contact at every location. The parameter values used are as in Table 4.

**Figure 9:**
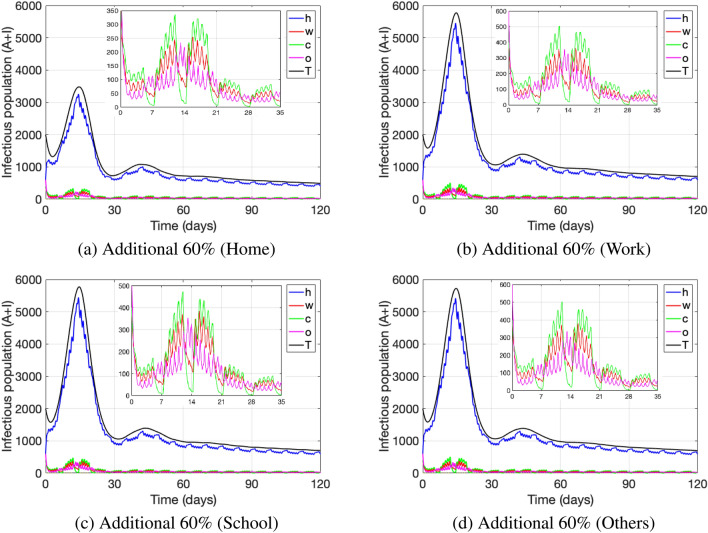
Addition (in percentage) to the transition rate leading to quarantine upon a close contact at a specific location. The parameter values used are as in Table 4.

Figures 8 and 9 show point prevalence levels at each location, when quarantine measures of infectious individuals and of non-infectious due to close contacts with infected are more efficiently enforced. That efficiency is represented by an increase in the rates $\psi _y$ and $\psi _y^i$, $y\in \{h,w,c,o\}$, representing the transition rates to quarantine at each location: home (*h*), work (*w*), school (*c*), other (*o*), either due to a close contact with an infected at the given location or due to being infectious. Figure 8 depicts an increase of 20%, 40% and 60% in all transition rates to quarantine. As expected, we observe that the higher that increase is, the less is the point prevalence level of the disease overall (black curve) and also at each location, when compared to the base line case. Hence, measures that make quarantine measures more efficient, such as stricter tracing of close contacts and enforcement of quarantines, reduce the number of active cases (point prevalence) significantly.

On the other hand, Fig. 9 depicts the effect of stricter close contact tracing and quarantine enforcement in only one location, when increasing the transition rates $\psi _y$ and $\psi _y^i$ to quarantine by 60% for (a) home, (b) work, (c) school and (d) other. As compared to the base line case depicted in Fig. 8, we observe that stricter quarantine enforcement in the home setting has the biggest effect on reducing the number of total active cases and especially cases at home, while reducing the active cases at other locations as well. On the other hand, we observe that stricter quarantine enforcement only at work, school or others decreases the number of active cases only slightly. These results enforce the importance of implementing strict quarantine measures in the home setting. We observe that strict close contact tracing in the home setting (friends and family) in order to implement quarantine measures is essential to control disease spread. Even if those strict measures are implemented at other locations such as work, school and other, it may not be enough to lower the number of active cases if they are not implemented in the home setting.

**Table 4:** Baseline numerical values occupied in the numerical simulations by blocks during the week and on weekends.

Parameters		Baseline numerical values in each time block						Ref.
	Day	I	II	III	IV	V	VI	
$\delta$	Weekday	0.022	0.0645	0.3585	0.7095	0.7235	0.6060	^41^
	Weekend	0.3	0.01	0.1	0.1	0.3	0.2	
$\delta ^{i}$	Weekday	0.022/2	0.0645/2	0.3585/2	0.7095/2	0.7235/2	0.6060/2	Ac
	Weekend	0.3/2	0.01/2	0.1/2	0.1/2	0.3/2	0.2/2	
$\alpha _{w}=\alpha _{w}^{i}$	Weekday	0.5	0.5	0.45	0.4	0.4	0.1	^41^
	Weekend	0.2	0.1	1	0.3	0.1	0.2	
$\alpha _{c}=\alpha _{c}^{i}$	Weekday	0.3	0.0	0.45	0.4	0.4	0.3	^41^
	Weekend	0.0	0.0	0.0	0.3	0.0	0.0	
$\alpha _{o}=\alpha _{o}^{i}$	Weekday	0.2	0.5	0.1	0.2	0.2	0.6	^41^
	Weekend	0.8	0.9	0.0	0.4	0.9	0.8	
$\sigma _{w}$	Weekday	3/4	3/4	3/4	3/4	3/4	3/4	^41^
	Weekend	3/4	3/4	3/4	3/4	3/4	3/4	
$\sigma _{c}$	Weekday	4/4	4/4	4/4	4/4	4/4	4/4	^41^
	Weekend	2/4	2/4	2/4	2/4	2/4	2/4	
$\sigma _{o}$	Weekday	2/4	2/4	2/4	2/4	2/4	2/4	^41^
	Weekend	3/4	3/4	3/4	3/4	3/4	3/4	
$\lambda _{1}$	Weekly	0.7	0.7	0.7	0.7	0.7	0.7	^39^
$\lambda _{2}^{h}$	Weekday and weekend	0.5	0.5	0.5	0.5	0.5	0.5	Ac
$\lambda _{2}^{w}$	Weekday and weekend	0.4	0.4	0.4	0.4	0.4	0.4	Ac
$\lambda _{2}^{c}$	Weekday and weekend	0.3	0.3	0.3	0.3	0.3	0.3	Ac
$\lambda _{2}^{o}$	Weekday and weekend	0.2	0.2	0.2	0.2	0.2	0.2	Ac
$D_{h}^{*}$	Weekday and weekend	0.53	0.53	0.53	0.53	0.53	0.53	^33^
$D_{w}^{*}$	Weekday and weekend	1.25	1.25	1.25	1.25	1.25	1.25	^33^
$D_{c}^{*}$	Weekday and weekend	0.83	0.83	0.83	0.83	0.83	0.83	^33^
$D_{o}^{*}$	Weekday and weekend	1.65	1.65	1.65	1.65	1.65	1.65	^33^
$\psi _{h}$	Weekday and weekend	$3.08*0.75$	$3.08*0.75$	$3.08*0.75$	$3.08*0.75$	$3.08*0.75$	$3.08*0.75$	^22^
$\psi _{w}$	Weekday and weekend	$2.81*0.35$	$2.81*0.35$	$2.81*0.35$	$2.81*0.35$	$2.81*0.35$	$2.81*0.35$	^22^
$\psi _{c}$	Weekday and weekend	$1.88*0.50$	$1.88*0.50$	$1.88*0.50$	$1.88*0.50$	$1.88*0.50$	$1.88*0.50$	^22^
$\psi _{o}$	Weekday and weekend	$5.63*0.40$	$5.63*0.40$	$5.63*0.40$	$5.63*0.40$	$5.63*0.40$	$5.63*0.40$	^22^

*Ac* author chosen.

## Discussion and conclusions

Social science support in the field of human behavior is needed to align with epidemiological recommendations and to better understand the evolution of COVID-19. In this respect, different perspectives have been presented by the authors in^37^ for identifying insights regarding social and behavioral approaches during the current pandemic. Such findings show that a strong fear might change the behavior of the people if they feel a sense of efficacy in the measure taken, but nevertheless also a bias optimism among certain people can be observed.

Consequently, depending of their perception on health risk, individuals might return to normal life by relaxing their protective measures from increased social distancing to natural distancing and contacting behaviors, providing favorable conditions for disease transmission^40^. Some studies have even shown evidence of dispositional resistance to change across different countries^40–42^, revealing cultural differences amongst groups of people in term of strong or weak resistance to change. To account for changes in distancing behavior, our model includes a dynamic resistance- and fear-dependent distancing behavior that affects disease transmission, and specifically, our results show the importance of distancing behavior in the home setting that is essential even under strict mobility restrictions (see Fig. 7). In particular, increasing the natural distance in the home setting may decrease significantly the number of active cases overall. In practice, reducing the number of visits from relatives and/or friends, avoiding long time exposure with them and also preventing crowded gatherings, can serve as handy measures to prevent infection. These findings reveal the importance of promoting family awareness through social distancing as a way to mitigate COVID-19 transmission, even while mobility to work, school and other activities may be restricted and social distancing there may be mandatory.

Another critical issue that affects disease transmission is how people spend their time and where they gather. Evidence suggests that time spent at different locations (e.g., work, school, other leisure activities, etc.) also depends on cultural practices^43,44^. A multicultural study conducted by the University of Oxford revealed information referred to paid work and study hours distributed per week and weekends^44^, using an extensive data set that included information from Belgium, Finland, Germany, Italy, Norway, Spain, Sweden, Bulgaria, Estonia, Latvia, Lithuania, Poland, Slovenia, Japan, Brazil, and United States. In addition, the American Time Use Survey (ATUS)^43^ determined how much time individuals in the United States spend on various activities, such as home activities, work, educational, and leisure activities. Also, a report made by Data Quality^45^, detailed the scheduled time Americans spend on different activities throughout the day (24 h), via an infographic picture. Finally, a recent platform for local mobility was searched to complete a set of information required to better understand people’s mobility, clustered by the type of activities that different communities around the world established to combat COVID-19^45^. This information is obtained when comparing with baseline pre-pandemic mobility indices.

Based on information from the previously mentioned studies, we structured our model including home, school, work and other location settings; and considered mobility from home to these settings. Our results show that in general restricting mobility from home to only one location does not impact significantly the number of active cases overall, each measure does though reduce the cases at that specific location. In particular, we observe that restricting school attendance reduces the transmission rate at home the most, especially during weekdays, but not during weekends. We observe a similar but less pronounced effect when restricting work attendance. Restricting other leisure activities reduces home transmission specifically at certain time blocks during weekends. These results show again the importance of the transmission in the home setting (which is the highest among locations), and how measures restricting the mobility from home to only one individual location may not be enough to control the disease overall, since under such a restriction, transmission at home may remain high at certain times during the week (see Fig. 4). Hence, a combination of restrictive measures needs to be considered to really reduce disease transmission. Indeed, we present simulations considering several restrictive measures applied at once, which produce a larger impact on case reduction overall (see Fig. 5). Our results show that if simultaneously school and work attendance is reduced, the most reduction in home transmission during the day on weekdays can be observed, but still remaining high during weekends, unless mobility to other locations is restricted as well. This shows the importance of complementing with other type of protective measures, such as keeping social distance or mask use to reduce overall transmission. Social distancing shows to be particularly important during weekend activities with family and friends (home setting), which indeed has a large impact according to Fig. 7 as discussed before. Therefore, our results conclude that safety at work and school—either by implementing online activities (keeping people at home) or social distancing—may not be enough to reduce active cases to a desired level, if safety measures in the home setting are neglected.

Another important safety measure to prevent disease spread is quarantining individuals after being in close-contact with an infectious person or isolating infectious individuals^88^. It is worth to highlight that, whether a non-infected person must go into quarantine depends on the definition of close-contact and how well in practice this definition is implemented. We incorporated classes of quarantined individuals, which get there either through the infectious class (go into isolation) or due to having been in close contact with an infectious. Our results show that, when quarantines due to close-contact with an infectious are more efficiently enforced and strict contact tracing is implemented, a significant decrease in infectious cases can be observed. We can see this effect especially if this is done in the home setting, while stricter quarantine enforcement only at work or school just slightly reduces the number of cases overall. This highlights the importance of strictly following and eventually hardening the close-contact definition, especially at home, in order to control the disease.

The new complexity on the transmission of infectious diseases has lead us to incorporate human interactions and reactions to improve the modeling course of the epidemics^18^. Scientific evidence has highlighted changes in the mechanisms of transmission of this novel SARS-CoV-2, when individuals modify their behavior^48,51^. Likewise a similar approach has been implemented for modelling the spread of other infectious diseases^48,75^ targeted to propose more effective control measures to mitigate the spread of the disease. Also, it has been previously suggested to incorporate social, behavioral factors within mathematical disease models to better understand how personal interactions in a society affect disease transmissions; in particular physical contact, social distancing behavior, and social mapping of activities^38^. We believe that our work contributes to the existing literature by including these aspects in a novel way, implementing the simple idea presented in^54^—that connects disease transmission with social distancing—, while incorporating it into a location-dependent and time-block-dependent structure, essential for mobility of individuals.

In general, the strength of the present study is the integration of multiple mobility scenarios based on a mathematical approach of Susceptible-Exposed-Asymptomatic-Infectious-Recovered-Susceptible model, with quarantine and social distance dependent transmission rates to study COVID-19 dynamics. Nevertheless, our model shows the limitations intrinsic to extended SIR type models, and is not an exception when considering the difficulties that need to be overcome when analyzing model uncertainty. Before fitting the model to real data, much work needs to be done in conjunction with human behavior scientists, epidemiologists and statisticians, to better understand human behavioral factors used in our model and to obtain data regarding these. Future work entails an interdisciplinary study of this kind. Finally, our model allows to study a variety of potential scenarios using combination of parameters (such as, the ones related to mobility restrictions, average time spent at each location, distribution of activities, cultural factors, close contact definition, etc.), which provides a vast framework for future studies connecting human behavioral factors and disease transmission.
